# Ultrasound and microbubble induced release from intracellular compartments

**DOI:** 10.1186/s12896-017-0364-3

**Published:** 2017-05-18

**Authors:** Farah Hussein, Costin Antonescu, Raffi Karshafian

**Affiliations:** 1 Department of Physics, Ryerson University, 350 Victoria Street Toronto, Ontario, M5B 2K3 Canada; 2 Department of Chemistry and Biology, Ryerson University, Toronto, Ontario Canada; 3 Institute for Biomedical Engineering, Science and Technology (iBEST), Toronto, Canada; 4 Keenan Research Centre, St. Michael’s Hospital, Toronto, Canada

**Keywords:** Ultrasound and microbubble, Sonoporation, Endocytosis, Exocytosis, Intracellular uptake, Intracellular release, Acoustic cavitation bioeffects, In vitro ultrasound bioeffects, Cellular bioeffects of ultrasound

## Abstract

**Background:**

Ultrasound and microbubbles (USMB) have been shown to enhance the intracellular uptake of molecules, generally thought to occur as a result of sonoporation. The underlying mechanism associated with USMB-enhanced intracellular uptake such as membrane disruption and endocytosis may also be associated with USMB-induced release of cellular materials to the extracellular milieu. This study investigates USMB effects on the molecular release from cells through membrane-disruption and exocytosis.

**Results:**

USMB induced the release of 19% and 67% of GFP from the cytoplasm in viable and non-viable cells, respectively. Tfn release from early/recycling endosomes increased by 23% in viable cells upon USMB treatment. In addition, the MFI of LAMP-1 antibody increased by 50% in viable cells, suggesting USMB-stimulated lysosome exocytosis. In non-viable cells, labeling of LAMP-1 intracellular structures in the absence of cell permeabilization by detergents suggests that USMB-induced cell death correlates with lysosomal permeabilization.

**Conclusions:**

In conclusion, USMB enhanced the molecular release from the cytoplasm, lysosomes, and early/recycling endosomes.

**Electronic supplementary material:**

The online version of this article (doi:10.1186/s12896-017-0364-3) contains supplementary material, which is available to authorized users.

## Background

The efficiency of drug therapy partly depends on the amount of drug delivered to the target cell and the release of the drug from the cell [1]. Uptake and release of molecules in cells can occur through intrinsic cellular processes such as endocytosis and exocytosis, respectively [2, 3], as well as through intrinsic membrane transporters for specific molecules such as glucose or ions [2]. The intracellular delivery of macromolecules can be enhanced through the application of ultrasound and microbubbles (USMB) by inducing plasma membrane disruption [4–7] and enhancing endocytosis [8–10]. In addition, USMB can also induce the release of cytoplasmic content and exocytosis of lysosomes immediately following exposure [11–13]. However, the bioeffects on the lysosomes that remain inside the cell without fusing with the plasma membrane and the release from other membrane bound cellular compartments are still unknown. In addition, the effect of time on the cellular release of molecules following USMB is not known.

Studies have shown that USMB can enhance the intracellular uptake of molecules, which otherwise would be excluded from cells, through mechanically inducing pore-like plasma membrane disruption in a phenomenon known as sonoporation [4, 5, 14]. In addition, USMB enhances the intracellular uptake of molecules through endocytosis, which is an active mechanism of molecular uptake in biological cells [8]. Molecules that enter a cell through membrane disruptions are localized in the cytoplasm, while molecules that enter through endocytosis are initially localized within membrane bounded vesicles [8, 10]. These vesicles can be transported to recycling endosomes, where some proteins and receptors are recycled back to the cell surface or subjected to membrane traffic to lysosomes for degradation [15]. The USMB enhanced endocytosis may be associated with the mechanism of membrane repair, perhaps resulting in internalization of the damaged part of the plasma membrane, as is known to occur in response to bacterial pore-forming toxins [16]. The dominant mechanism of enhanced uptake may be partly dependent on the size of the delivered molecules [8].

USMB may induce the release of cellular contents into the extracellular space by diffusion through disruptions on the plasma membrane, as shown by the release of hemoglobin from human erythrocytes [12] and Green Fluorescent Protein (GFP) from GFP-transfected HeLa cells [11] immediately following exposure to USMB. However, the release of the cytosolic indicator in these studies did not differentiate between viable and non-viable cells following exposure. As non-viable cells are expected to exhibit disruption of membrane integrity, distinguishing the release of material from viable *vs*. non-viable cells is an important consideration. In addition, USMB can induce lysosomal exocytosis immediately following exposure, triggered by the influx of Ca^2+^ ions through the membrane disruptions [13]. However, the broader bioeffects of USMB on endomembrane traffic, including the effects on recycling or plasma membrane fusion of intracellular lysosomes and early/recycling endosomes, thus controlling release of their contents is not well understood. This study examines the effects of USMB on the release of molecules from specific cell compartments, including the cytoplasm, lysosomes, and early/recycling endosomes up to 21.5 min following exposure. The hypothesis guiding this study is that USMB can enhance the release of molecules from cells through membrane disruption and enhanced exocytosis. Experiments were conducted in vitro using Retinal Pigmented Epithelial (RPE) cells that were treated with USMB and analyzed using flow cytometry and microscopy. The results obtained from this study have demonstrated that USMB enhanced the release of molecules from cells.

## Methods

The effects of USMB on the release of molecules from early/recycling endosomes, cytoplasm, and lysosomes at 1.5 min, 11.5 min, and 21.5 min following the start of USMB were examined. This was done by tracking either Alexa647–Transferrin, GFP signal in RPE cells stably transfected with a soluble GFP-tagged protein (clathrin), and lysosomal associated membrane protein-1 (LAMP-1) using specific antibodies, respectively [17, 18]. Notably, soluble transferrin is a well-established marker of the early and recycling endosomes, as is LAMP-1 of the late endosome/lysosomes and these two markers are mostly non-overlapping in diverse types of cells [19–22].

### In vitro cell model

RPE cells, originally obtained from ATCC, were exposed to USMB, with cells either in suspension or monolayer configurations. The cells were cultured in tissue culture flasks under 5% CO_2_ at 37 °C in Ham’s DMEM F12 medium, (Life technologies, Carlsbad, CA), supplemented with 10% (v/v) fetal bovine serum and antibiotics (10 mg/mL streptomycin and 66 μg/mL penicillin) and harvested by trypsinization. With the exception of microscopic studies of monolayers, the majority of the experiments described here were flow cytometric studies of cells treated while in suspension and fixed prior to flow cytometry. Cells were re-suspended at 10^6^ cells/mL and treated with USMB in suspension. For cell monolayer studies, cells were seeded at 50% confluence on glass coverslips in 6-well plates for 24 h and subsequently treated with USMB.

Cells in suspension were treated with USMB and the release of molecules from cells was assessed using fluorescent markers and flow cytometry. The release of molecules was examined at 37 °C at 1.5, 11.5 or 21.5 min following the start of USMB. These time delays would be sufficient to observe the fast release from the lysosomes and the cytoplasm and the relatively slower release from early/recycling endosomes [13, 23]. Independent samples of cells were used to investigate the release of fluorescent markers from three cell compartments: (1) early/recycling endosomes, (2) cytoplasm, and (3) lysosomes for each time point. The earliest time point was 1.5 min which corresponds to 60 s of USMB treatment and 30 s of handling time until the samples are transferred on ice to stop the release of molecules. Subsequently, the cells were fixed with 4% PFA prior to flow cytometry analysis. The relative mean fluorescent intensity (MFI) of each fluorescent marker was measured using flow cytometry to indicate the amount of marker in cells treated or not with USMB. In addition, GFP-transfected RPE (RPE-GFP) cells (GFP fused to the cytosolic protein clathrin) were treated with USMB in a monolayer and the localization of bound LAMP-1 antibody at 1.5 min along with changes in GFP MFI were examined using confocal fluorescence microscopy.

### Release from early/recycling endosomes

The release from early/recycling endosomes was investigated by loading RPE cells with 79 kDa Alexa647-Transferrin (Tfn) [24, 25]. Cells in suspension were loaded with Tfn by incubating with DMEM serum starvation media (D5796., Sigma-Aldrich Co., Oakville, ON, CA) for 60 min, replacing the starvation media with PBS^3+^ (Phosphate Buffered Saline containing 1 mM MgCl_2_, 1 mM CaCl_2_, and 5 mM glucose) containing 20 μg/mL Tfn, and allowing the cells to internalize Tfn for 60 min at 37 °C. Excess Tfn was removed by centrifugation at 900 rpm for 5 min at 4 °C and washing with 1 mL ice-cold PBS two times. Then, the cells were suspended in 37 °C PBS^3+^, exposed to USMB, and transferred to 4 °C to stop the release of the marker at each time point. Subsequently, the cells were washed with ice-cold PBS to remove the released marker, stained with a viability stain, and fixed to be analyzed. The MFI of Alexa647, which indicates the amount of Tfn inside the cell, was measured in untreated and USMB-treated samples.

### Release from the cytoplasm

RPE-GFP cell samples were exposed to USMB, transferred to 4 °C at each time point, washed twice with ice-cold PBS to remove any released GFP, then stained with a viability stain, and fixed. After fixation, the MFI of GFP was measured to indicate the amount of GFP in the cytoplasm of the cell in untreated and USMB-treated samples.

### Release from lysosomes

The release of lysosomal content was assessed using a Phycoerythrin (PE)-conjugated antibody against the lumemal/extracellular domain of LAMP-1 (A15798, Life technologies, Carlsbad, CA). Cells in suspension were treated with USMB then transferred to 4 °C to stop the release at each time point. The samples were washed with ice-cold PBS to remove any released material and transferred back on ice, stained with a viability stain, and fixed. The fixed cells were stained with LAMP-1 antibody (400 μL/mL) for 30 min at room temperature. Unbound antibody was removed by washing three times with PBS. The MFI of LAMP-1 antibody was measured using flow cytometry to indicate the amount LAMP-1 antibody binding. Increased LAMP-1 antibody binding indicates exposure of the lumen of lysosomes to the antibody which indicates either 1) lysosomal fusion with the cell membrane or 2) the permeabilization of both the plasma membrane and intracellular lysosomes.

### Cell viability

Cell viability in the untreated control and in USMB-treated samples was measured using 7-Aminoactinomycin D (7-AAD), (559925, BD Biosciences, Mississauga, ON, CA), in combination with all of the markers before fixation by incubating 5 μL of 0.5 mg/mL 7-AAD with the cell samples on ice for 30 min. After incubating with 7-AAD, the 7-AAD was removed from the medium by washing three times with ice-cold PBS. Then, the cells were fixed with 4% PFA in PBS for 15 min at room temperature. The incorporation of 7-AAD indicates that the membranes of these cells were not able to recover over the time period [26, 27]. Cells that do not incorporate 7-AAD were considered viable cells, and 7-AAD positive cells were considered non-viable cells.

### The localization of bound LAMP-1 antibody in the cell

GFP-RPE cells in a monolayer were treated with USMB then placed on ice at 1.5 min from the start of the USMB treatment. The samples were washed twice with ice-cold PBS to remove any released cellular material, fixed with 4% PFA in PBS for 15 min then stained with LAMP-1 antibody (400 μL/mL) for 30 min at room temperature. The coverslips were then mounted on slides and imaged with confocal fluorescence microscopy using a Leica TCS SL microscope (Leica TCS SL., Leica Microsystems Inc., Wetzlar, Germany) at 40× magnification.

### The subcellular localization of Tfn and LAMP-1

RPE cells not expressing any fluorescent protein (parental RPE cells) in a monolayer were treated with 20 μg/mL Alexa555-conjugated transferrin (A555-Tfn) for 60 min at 37 °C. The samples were washed twice with ice-cold PBS to remove any excess (unbound) A555-Tfn, fixed with 4% PFA in PBS for 15 min and then permeabilized and stained with LAMP-1 antibody (400 μL/mL) for 60 min at room temperature. The coverslips were then mounted on slides and imaged using epifluorescence microscopy. The widefield epifluorescence microscopy images presented in Additional file 1: Figure S1 were obtained using a 63× (NA 1.49) oil objective on a Leica DM5000 B epifluorescence microscope using a DFC350FX camera (Leica DM5000 B., Leica Microsystems, Wetzlar, Germany). Indeed, Transferrin and LAMP-1 labeled two largely distinct membrane-bound intracellular compartments in the RPE cells used in this study (Additional file 1: Figure S1).

### USMB exposure

**Fig. 1:**
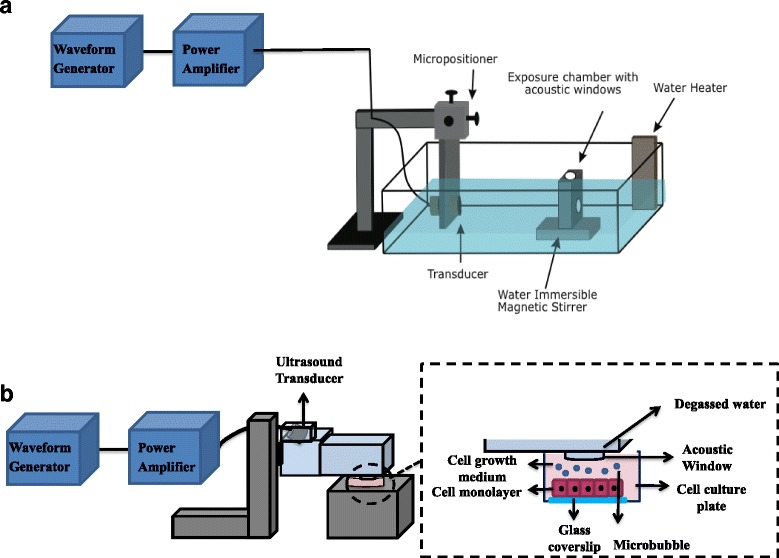
**a** In-suspension ultrasound and microbubble exposure setup. It consists of a waveform generator connected to a power amplifier that sends the signal to a 500 kHz ultrasound transducer that is focused on an acoustic window in the treatment chamber using a micro-positioning system. The sample chamber is placed on a magnetic stirrer. **b** Monolayer USMB exposure setup. It consists of a waveform generator connected to a power amplifier that sends the signal to a 500 kHz ultrasound transducer which is focused on an acoustic window where the sample is placed

Cells were exposed to 500 kHz pulse centre frequency, 570 kPa peak negative pressure (Pneg), 32 μs pulse duration (16 cycles tone burst) at 1 kHz pulse repetition frequency (PRF) corresponding to 3.2% duty cycle, for 60 s in 37 °C PBS^3+^. Immediately prior to ultrasound treatment of each sample, Definity mirobubbles (Lantheus Medical Imaging Inc., Saint-Laurent, QC, CA) were added. The Definity microbubbles were activated using a Vialmix for 45 s. Using the cell suspension model, cells at 10^6^ cells/mL were exposed to ultrasound using a single element focused transducer of 500 kHz frequency, 9.2 mm −6 dB beam width, and 50 mm focal distance (IL0508HP, Valpey Fisher Inc, Hopkinton, MA, USA). A schematic diagram of the USMB exposure setup is shown in Fig. 1 (a). The setup consisted of an arbitrary waveform generator, connected to a power amplifier (AG series Amplifier, T&C power conversion, Inc., NY, USA), which transmitted the electrical signal to the ultrasound transducer; the transducer was focused at the centre of the treatment chamber. A volume of 10 μL of microbubbles was added to the 0.6 mL cell sample in the treatment chamber and a magnetic stirrer assured the mixing of cells and bubbles during the treatment. Using the monolayer model, cells were exposed to USMB using a 500 kHz single element flat transducer with 32 mm element diameter focused at 85 mm and a -6 dB beam width of 31 mm at the focal point (IL0509GP., Valpey-Fisher Inc., Hopkinton, MA, USA) at a microbubble concentration of 10 μL/mL. The wells of the six-well plates were filled with 13 mL PBS^3+^. A schematic diagram of the monolayer USMB exposure setup is shown in Fig. 1 (b). The setup consisted of an arbitrary waveform generator, connected to a power amplifier (AG series Amplifier, T&C power conversion, Inc., NY, USA), which transmitted the electrical signal to the ultrasound transducer. The transducer was submerged in partially degassed deionized water and focused obliquely at the centre of an acoustic window.

### Analysis

A MACSQuant flow cytometer (MACSQuant® Instrument., Miltenyi Biotec GmbH, Bergisch Gladbach, Germany) was used in this study. A 488 nm laser was used to excite GFP, PE and 7-AAD and 638 nm laser was used to excite Alexa-647. The laser powers were 30 mW and 20 mW for the 488 nm and the 638 nm lasers, respectively. Fluorescence emitted from GFP was collected using a 525/50 nm bandpass filter while a 585/40 nm bandpass filter was used for collecting PE fluorescence. A 750LP bandpass filter was used for 7-AAD and a 655–730 nm bandpass filter was used for Alexa-647. The instrument was configured for medium sample flow rate (50 μL/min). Data were acquired and analyzed using Macsquantify software. At the beginning of every acquisition session, a gate was set using a negative cell control to select single cell events. Of the single cell population, gates were set around the sub-populations of interest where the percentage of cells and the mean fluorescent intensity in each gate was recorded. In every experiment, a negative control of unstained RPE cells was used to identify the cell population in addition to a 7-AAD stained control of untreated cells. In addition, a positive control of 7-AAD stained cells was prepared by adding 7-AAD to cells permeabilized by fixation with PFA. In the analysis, single stained cell samples with Tfn-Alexa-647, GFP and LAMP-1 antibody were used. The MFI values of the markers in 10,000 cells from each sample were measured and were normalized to the MFI values for the untreated control at t = 1.5 min. The number of samples was *n =* 4-6 and Mann–Whitney U non-parametric test was used to indicate statistically significant differences in MFI between groups. The MFI values of the USMB-treated groups were considered significantly higher than the untreated groups if the *p-*values associated with the one-tailed Mann–Whitney *U* test were less than a significance level of α = 0.05. In addition, the MFI of GFP and LAMP-1 antibody from the fluorescent images acquired with the monolayer configuration were measured using ImageJ. Sixty cells from three independent trials per condition were examined from each group. The experimental conditions and the number of independent samples per condition are shown in Table 1.

**Table 1:** Experimental conditions and number of independent samples per condition

	1.5 min/no USMB	11.5 min/no USMB	21.5 min/no USMB	1.5 min/USMB	11.5 min/USMB	21.5 min/USMB
Tfn +7-AAD	*n =* 4	*n =* 4	*n =* 4	*n =* 4	*n =* 4	*n =* 4
GFP + 7-AAD	*n =* 4	*n =* 4	*n =* 4	*n =* 6	*n =* 6	*n =* 6
LAMP-1 + 7-AAD	*n =* 4	*n =* 4	*n =* 4	*n =* 4	*n =* 4	*n =* 4

## Results

The biological effects of ultrasound and microbubble treatment, in particular with respect to the induction of release of cellular material by this treatment, remain poorly understood. To determine how USMB treatment impacts cellular release of material from the cytoplasm, endosomes and lysosomes, we have used specific markers of each compartment and assessed the release of each marker from cells upon exposure to USMB.

### Cell viability

**Fig. 2:**
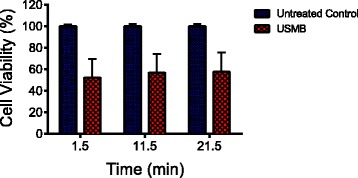
The percentage of viable cells in ultrasound and microbubble (USMB) treated and untreated control at 1.5, 11.5, and 21.5 min assessed using 7-Aminoactinomycin D (7-AAD). The number of samples is *n =* 12 for the untreated group and *n =* 14 for the USMB treated group. The error bars represent the standard deviation

Figure 2 shows the percentage of viable cells in USMB-treated and untreated control samples at 1.5, 11.5 and 21.5 min after the start of USMB exposure normalized to the cell viability of the untreated control at 1.5 min. As expected, USMB induced a decrease in cell viability. The percentage of viable cells decreased from 100 ± 2% in the untreated control to 55 ± 17% in the USMB-treated group at 1.5 min. The percentages of viable cells in both the untreated controls and USMB-treated samples remained unchanged over the ~20 min duration; no statistically significant differences were observed.

### Release from early/recycling endosomes

**Fig. 3:**
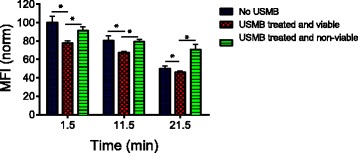
The mean fluorescent intensity (MFI) in untreated controls and ultrasound and microbubble (USMB) treated Alexa647-Transferrin (Tfn) loaded cells at 1.5, 11.5, and 21.5 min. The number of samples is *n =* 4 for all groups and the (*) indicates statistical significance (*p <* 0.05) using a Mann–Whitney *U* test. The error bars represent the standard deviation

Figure 3 shows the MFI corresponding to intracellular (endosomal) Tfn in untreated control cells and USMB-treated cells at 1.5, 11.5 and 21.5 min with respect to the start of USMB exposure; the data were normalized to the MFI of the untreated control at t = 1.5 min of Tfn recycling. In the control cells, the reduction of MFI represents the basal rate of TfR recycling as it is undergoing constitutive endocytosis and recycling. Importantly, USMB significantly increased Tfn release from early/recycling endosomes immediately (at 1.5 min) and the amount of intracellular Tfn remained lower than control up to 21.5 min in viable USMB-treated cells. The MFI of intracellular Tfn in USMB-treated viable cells decreased from 100% to 77%, from 80% to 67%, and from 50% to 46% at 1.5, 11.5 and 21.5 min, respectively. In contrast, the MFI corresponding to A555-Tfn in non-viable USMB-treated cells was not statistically significant compared to the untreated control.

### Release from cytoplasm

**Fig. 4:**
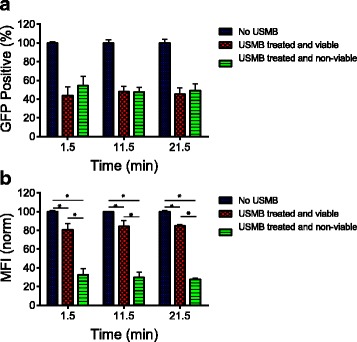
**a** The percentage of Green Fluorescent Protein positive (GFP+) cells measured in ultrasound and microbubble (USMB) treated and untreated groups at 1.5, 11.5, and 21.5 min. **b** The mean fluorescent intensity (MFI) in GFP+ cells in untreated and USMB treated cells at 1.5, 11.5, and 21.5 min. The (*) indicates that *p <* 0.05 using a Mann–Whitney *U* test. The number of samples is *n =* 4 for the untreated control and *n =* 6 for USMB treated group. The error bars represent standard deviation

Figure 4 (a) shows the percentage of GFP positive cells (GFP+) in untreated control and USMB-treated samples at 1.5, 11.5 and 21.5 min. USMB did not significantly affect the percentage of GFP+ cells after the treatment. All (100%) of the cells were GFP+ before USMB and a total of 97% (including viable and non-viable cells) remained GFP+ after USMB. Upon USMB exposure, of the 97% that remained GFP+, 45% of the cells were GFP+ and viable while 52% of the cells were GFP+ and non-viable. These percentages remained constant for ~20 min after USMB stimulation. Figure 4 (b) shows the MFI of GFP+ cells in untreated control and USMB-treated cells at 1.5, 11.5 and 21.5 min; the data were normalized to the MFI of the untreated control at 1.5 min. USMB caused an increase in the release of GFP from the cytoplasm, and the amount of GFP released was dependent on cell viability. The MFI decreased by 19% at 1.5 min in the viable USMB-treated group compared to the untreated control. In contrast to the small but statistically significant decrease in MFI of cytosolic GFP in viable cells, the MFI of cytosolic GFP in cells that became non-viable after USMB exposure decreased by robust 67% compared to the untreated control. The MFI decreased within 1.5 min from the start of USMB and did not significantly change with time. This indicates that upon USMB treatment, the majority of the loss of cytosolic material occurs in cells that are non-viable, while a smaller but significant release of cytosolic material occurs in cells that remain viable after USMB treatment. The effect of USMB on the release of cytosolic material from cells appears to be immediate (within 1.5 min) and does not significantly change with the time delay after USMB.

### Release from lysosomes

**Fig. 5:**
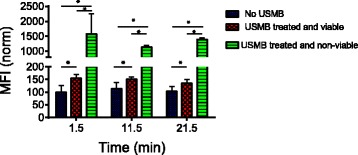
The mean fluorescent intensity (MFI) from Lysosomal Associated Membrane Protein-1. (LAMP-1) antibody binding measured in untreated control and ultrasound and microbubble (USMB) treated samples at 1.5, 11.5, and 21.5 min from the start of USMB. The (*) indicates a statistically significant difference (*p <* 0.05) using a Mann–Whitney *U* test. The number of samples *n =* 4 for both USMB treated and untreated groups. The error bars represent the standard deviation

Figure 5 shows the MFI of LAMP-1 antibody staining (without permeabilization by detergents) in untreated control and USMB-treated cells at 1.5, 11.5 and 21.5 min. All groups were normalized to the MFI of LAMP-1 antibody staining of the untreated control cells. USMB increased the binding of LAMP-1 antibody in cells as indicated by the increase in the MFI of the LAMP-1 antibody label in the USMB-treated cells at 1.5 min compared to the untreated control. However, this increase is highly dependent on cell viability. When compared to the untreated control, USMB induced a 50% increase in LAMP-1 antibody MFI in viable cells immediately after USMB. In stark contrast, USMB induced a dramatic increase in MFI in non-viable cells; the MFI increased by 15 fold compared to the untreated control. The USMB induced lysosome exocytosis (in viable cells) and the large increase in LAMP-1 antibody labeling (in non-viable cells) appeared to be immediate and remained constant over 21.5 min.

### Relationship between the release from cytoplasm and lysosomes

**Fig. 6:**
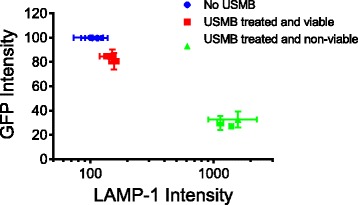
The relationship between the release from cytoplasm and lysosomes obtained by plotting the mean fluorescent intensities (MFI) of Green Fluorescent Protein (GFP) and Lysosomal Associated Membrane Protein-1 (LAMP-1) antibody. The MFI values for each marker were obtained from independent experiments and were normalized to the MFI values of the untreated control at t = 1.5 min for each marker. The error bars represent standard deviation

Figure 6 shows the relationship between the MFI of GFP and the MFI of LAMP-1 antibody in untreated and USMB-treated cells. The MFI values for each marker were obtained from independent experiments and normalized to the 1.5 min MFI values of the untreated control. There appears to be a correlation between the MFI of GFP (cytosolic marker) and the MFI of LAMP-1 antibody labeling in cells and this correlation appears to be dependent on cell viability. As the cell’s GFP MFI decreases, there is an increase in LAMP-1 antibody binding. A 19% decrease in GFP MFI in cells that remain viable after USMB is associated with a 50% increase in LAMP-1 antibody labeling. However, in non-viable cells, a 67% decrease in GFP MFI is associated with a 15-fold increase in LAMP-1 antibody labeling.

### The localization of binding LAMP-1 antibody in the cell

**Fig. 7:**
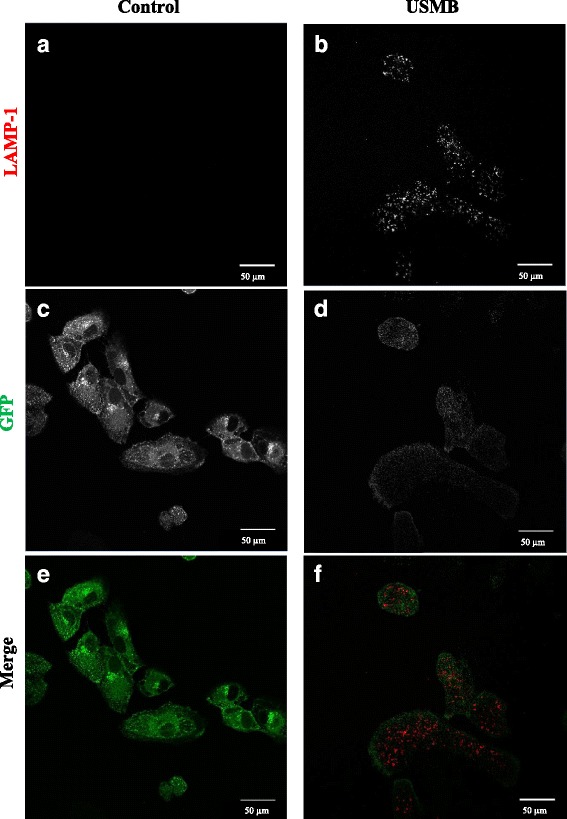
The localization of Lysosomal Associated Membrane Protein-1 (LAMP-1) antibody in Green Fluorescent Protein (GFP)-transfected Retinal Pigmented Epithelial cells (RPE). **a** LAMP-1 antibody binding in untreated control (**b**) LAMP-1 antibody binding in ultrasound and microbubble (USMB) treated cells (**c**) GFP-clathrin in untreated control (**d**) GFP-clathrin in USMB-treated cells (**e**) Merged channels of GFP-clathrin (*green*) and LAMP-1 antibody (*red*) in untreated control (**f**) Merged channels of GFP-clathrin and LAMP-1 antibody in USMB-treated cells (*n =* 3). Scale bar = 50 μm

The robust increase in LAMP-1 antibody labeling in cells that became non-viable upon USMB compared to that seen in cells that remained viable could be due to a much higher amount of lysosomal exocytosis in non-viable cells as these cells are attempting to repair their damaged membranes (within 1.5 min from the start of USMB), or could reflect partial permeabilization of lysosomal membranes (along with permeabilization of the plasma membrane) that correlates with loss of cell viability. To distinguish between these possibilities, the subcellular localization of LAMP-1 antibody staining was examined using immunofluorescence microscopy. Figure 7 shows the fluorescent images of cells expressing the cytosolic GFP marker and stained with LAMP-1 antibodies, as individual channels as well as their merged images, for untreated-control and USMB-treated cells. USMB-treated cells have significantly more LAMP-1 antibody labeling compared to the untreated control and this antibody labeling appears to be in well-defined punctate structures, consistent with LAMP-1 antibody labeling of intracellular structures, such as lysosomes, and not primarily on the cell surface (Fig. 7 (a) and (b)). Additionally, in the USMB treated cells, the labeling of cells with LAMP-1 antibody is highly heterogeneous, with some cells having more LAMP-1 antibody binding than others. Furthermore, cells that exhibit substantial LAMP-1 antibody staining appear to have lower GFP levels (cytosolic marker) compared to cells that exhibit lower levels of LAMP1 antibody staining, as shown in Fig. 7 (c) and (d). The merged images of LAMP-1 and GFP for untreated and USMB-treated cells are shown in Fig. 7 (e) and (f), respectively. The merged images show the dispersed distribution of GFP throughout the cytoplasm of the cell and the localization of LAMP-1 antibody in punctuate intracellular structures suggesting the permeabilization of intracellular lysosomes upon USMB exposure.

## Discussion

The efficacy by which USMB potentiates therapies involving the cellular uptake of biologically active molecules depends on the induction of enhanced uptake and minimal release from cells. In this study, it was demonstrated that USMB increased the release of fluorescent markers from specific cell compartments including the cytoplasm, lysosomes and early/recycling endosomes, and that this effect was highly dependent on cell viability. This could be an indication that the USMB-enhanced release of molecules is directly related to the membrane damage induced by USMB [11, 13]. USMB induced a decrease in cell viability and this is consistent with previous studies that show the effects of USMB on cell viability [6, 28]. This decrease in cell viability after USMB is the result of irreversible membrane disruption induced by USMB that allows 7-AAD to enter the cell and bind to the DNA [7].

Tfn release from early/recycling endosomes was significantly increased by USMB at 1.5 min in USMB-treated viable cells compared to the untreated control. However, this increase in recycling was not statistically significant in cells that were non-viable after USMB. Studies have suggested that the release of Tfn from endosomes is not driven by the increased calcium influx into the cell after membrane disruption [29]. Therefore, it can be suggested that the underlying mechanism of the increased release from early/recycling endosomes could be associated with an active, regulated cellular mechanism as a response to the biomechanical stress induced by USMB. This active cellular mechanism may be the result of enhanced endomembrane traffic flux, as USMB also enhances endocytosis, specifically clathrin mediated endocytosis which is the pathway of Tfn uptake or it may be in response to promoting plasma membrane repair [8–10]. Following internalization, Tfn is well known to populate several endosomal membrane compartments which have distinct recycling kinetics. For instance, recycling from an early endosomal subpopulation has t_1/2_ for recycling of ~ 2 min, while recycling from the perinuclear recycling endosomes has a t_1/2_ for recycling on the order of 10–20 min ([30]). The results of this study indicate that USMB may selectively trigger enhanced exocytosis from early, fast recycling endosomes. As such, after the initial burst of Tfn recycling in the 1.5 min following the start of USMB, Tfn recycling from these cells may be largely limited to that from perinuclear, slow recycling endosomal compartments. In contrast, cells not stimulated with USMB exhibit a constant rate of recycling from both fast and slow recycling endosomal compartments. This model readily explains why the recycling that occurs after the initial burst induced by USMB treatment in viable USMB-treated cells (in the period from 1.5 to 21.5 min) appears slower than in control cells, such that the difference in total cellular fluorescent transferrin decreases between control and USMB-treated cells over time. Additionally, the slower overall observed rate of Tfn recycling in cells which remained viable after USMB could be related to the oxidative stress induced by USMB through the production of reactive oxygen species (ROS) [31, 32] or due to depolarization and local hyperpolarization of the plasma membrane [33]. Furthermore, there was minimal release of Tfn in non-viable cells upon USMB treatment, in particular at 1.5 and 11.5 min following USMB treatment, indicating that USMB does not cause a non-specific permeabilization of intracellular membranes. Instead, non-viable cells largely retain Tfn intracellularly within endomembranes that fail to undergo exocytosis.

USMB induced the release from the cytoplasm of cells and this release was also found to be dependent on cell viability. Although USMB induced the release from the cytoplasm in both viable and non-viable cells, a much higher amount of cytoplasmic content was released from cells that became non-viable compared to cells that remained viable following USMB. Importantly, by comparing the release of cytoplasmic content in viable versus non-viable cells, this study identified that when measuring cytoplasmic release from cells upon USMB treatment, non-viable cells contribute much more to the measurement of cytoplasmic release than do viable cells. Hence, when studying the effects of USMB treatment on release of materials from the cytoplasm, it is important to distinguish between viable cells (that presumably rapidly close pores formed during sonoporation) and those that are non-viable (that fail to repair membrane damage caused by USMB treatment). This USMB-induced GFP release from the cytoplasm is consistent with the previously reported effect of USMB-induced release of GFP from the cytoplasm of the cell immediately following USMB exposure [11]. In addition, under our exposure conditions, the percentage of GFP+ cells remained consistent following USMB in contrast to a previous study where a decrease in GFP+ cells observed following USBM was concurrent with an increase in PI+ cells [11].

An increase in the detection of lysosomal content from the outside of the cell was observed after USMB and this increase was higher in non-viable cells compared to viable cells. In viable cells, this increase in detection of LAMP-1 likely reflects an increase in lysosome exocytosis upon USMB treatment. Cytosolic calcium levels were found to increase after sonoporation by the leaking of calcium into the cell with its concentration gradient through USMB-induced membrane disruptions [34]. The increased lysosomal exocytosis induced by calcium influx is due to the calcium-sensitive synaptotagmin VII membrane tethering protein on lysosomal membranes [13, 16]. Lysosomal exocytosis by complete fusion with the plasma membrane is thought to be the main mechanism of membrane repair after sonoporation [13]. There are two hypothesized mechanisms of membrane repair by lysosomal exocytosis: (1) lysosomes form a patch to repair the damaged site or (2) lysosomes deliver extra membrane to the cell surface to reduce membrane tension and help close the pores [13].

**Fig. 8:**
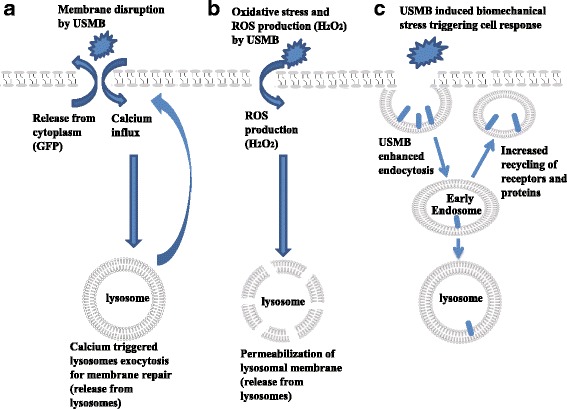
A schematic diagram summarizing the hypothesized mechanisms of USMB induced/enhanced release from cytoplasm, lysosomes, and early/recycling endosomes through both membrane disruption and exocytosis. These mechanisms can occur simultaneously and can affect the release from more than one compartment at the same time (**a**) Membrane disruption by USMB induces diffusion of molecules from the cytoplasm and causes an increase in Ca^2+^ influx which triggers lysosomes fusion for membrane repair and can also be involved in USMB enhanced endocytosis. **b** USMB induced production of H_2_O_2_ can induce the permeabilization of lysosomal membranes and the release of lysosomal content and this oxidative stress can also be involved in the slower rate of recycling from early/recycling endosomes (**c**) USMB induced biomechanical stress can cause enhancing endocytosis and triggering a cellular response driving release from early/recycling endosomes

In non-viable cells, the increased detection of lysosomal content from the cell exterior could be a result of exceptionally high levels of lysosomal fusion with the cell membrane as a mechanism for membrane repair [13, 35] or due to permeabilization of lysosomal membranes inside the cell [36]. That non-viable cells detected after USMB exhibit a large increase of punctate lysosomal signal and not a uniform signal provides evidence for the latter. Since the plasma membrane permeabilization induced by USMB is sufficient to allow efflux of small proteins, such as GFP, but not of organelles like lysosomes, permeabilization of both the plasma membrane and the lysosomes are needed in order for LAMP-1 antibody to access the lumen of lysosomes. This permeabilization of lysosomes in non-viable cells appears to be selective to this organelle, since a similar permeabilization of early/recycling endosomes was not observed in this cell population (as seen by the intracellular retention of transferrin in non-viable cells upon USMB treatment). Hence, it is unlikely that the increased permeabilization of lysosomes following USMB treatment merely reflects loss of plasma membrane integrity upon loss of cell viability, but instead may indicate that the lysosomal membranes may also be subject to permeabilization upon USMB. It is possible that excessive pore formation in lysosomes upon USMB treatment may contribute to loss of cell viability in some cells. The permeabilization of lysosomal membranes could be a result of oxidative stress generated by cellular treatment with USMB. Under the exposure conditions of this study which corresponds to a mechanical index (MI) of 0.8, the acoustic mechanism associated with enhanced leakage is inertial cavitation. Microbubbles exposed to high acoustic pressures and low pulse centre frequencies undergo inertial cavitation [37]. In addition, inertial cavitation has been shown to increase the temperature locally resulting in breaking of chemical bonds and the production of ROS including hydrogen peroxide (H_2_O_2_) [34]. Oxidative stress as a result of increased intracellular levels of H_2_O_2_ was found to induce permeabilization of lysosomal membranes inside the cell and this disruption of lysosomes is thought to be a signal for cell death [36]. Figure 8 summarizes the hypothesized mechanisms of USMB induced or enhanced release from cytoplasm, lysosomes, and early/recycling endosomes through both membrane disruption and exocytosis.

There are several limitations associated with the present study. The study did not examine the effects of varying treatment conditions such as microbubble concentrations and ultrasound pressure on the USMB enhanced release. Furthermore, it was assumed that ultrasound exposure without microbubbles has a minimal effect on the release of molecules from cells [11]. In addition, the 7-AAD viability marker stains cells whose membrane integrity has been compromised and does not assess the cell’s ability to proliferate and form a colony. However, this study improved our understanding of the limitations of USMB drug therapy, as it showed the effect of USMB on drug retention in cells. Additionally, the effect of USMB on the release of cellular content may shed the light on new applications of USMB as a possible way of extracting biologically active molecules from cells to further study them [11] or as a tool to increase the release of biomarkers from cancer cells to the blood stream for better cancer detection and localization [37, 38].

## Conclusions

USMB enhanced the release of molecules from early/recycling endosomes, cytoplasm and increased access to lysosomal content from the cell exterior through both membrane disruption and enhanced exocytosis. USMB enhanced the release of molecules in viable and non-viable cells to differing extents. A higher release of molecules from the cytoplasm and access to lysosomal content was observed in cells that were non-viable after USMB compared to cells that remained viable after the treatment and to the untreated control, suggesting that excessive release of cytoplasm or access to lysosomal content may be related to cell death upon USMB treatment. In contrast, a higher release of the contents from early/recycling endosomes was observed in viable cells compared to USMB-treated non-viable cells and to the untreated control, indicating that increased endomembrane recycling upon USMB was due to regulated, active processes stimulated by this treatment. In addition, the USMB induced release from the cytoplasm and extracellular access to lysosomal content appears to be immediate and independent of the time delay following USMB. Furthermore, LAMP-1 antibody labeling suggested the selective disruption of lysosomal membranes inside the cell after USMB treatment.

## Additional file

Additional file 1: Figure S1. Transferrin-loaded endosomes and LAMP-1 positive lysosomes are largely distinct compartments. RPE cells (not expressing any endogenous fluorescent proteins) were incubated with 10 μg/mL Alexa-555 conjugated transferrin (A555-Tfn) for 1 h at 37C. Subsequently, cells were rapidly washed, fixed, permeabilized, and subjected to immunofluorescence staining to detect endogenous LAMP-1. Cells were then further processed and subjected to widefield epifluorescence microscopy. Shown are two sets of representative micrographs of cells (under identical conditions) showing A555-Tfn (*red*), LAMP-1 (*green*), DAPI (*blue*) and merged images. These images showing little appreciable overlap of LAMP-1 and A555-Tfn signals. Scale = 10 μm. (PDF 4254 kb)

## Abbreviations

7-AAD: 7-Aminoactinomycin D
A555-Tfn: Alexa555-conjugated transferrin
GFP: Green fluorescent protein
GFP+: Green fluorescent protein positive cells
LAMP-1: Lysosomal associated membrane protein-1
MFI: Mean fluorescent intensity
MI: Mechanical index
PE: Phycoerythrin
P_neg_: Peak negative pressure
PRF: Pulse repetition frequency
ROS: Reactive oxygen species
RPE: Retinal pigmented epithelial cells
Tfn: Alexa646-Transferrin
USMB: Ultrasound and microbubbles
