# Supplementary figures and images for: Ultrasound and microbubble induced release from intracellular compartments
Source: BMC Biotechnol. 2017 May 18;17:45. doi: 10.1186/s12896-017-0364-3 (PMC5437622; doi:10.1186/s12896-017-0364-3)

Figure S1

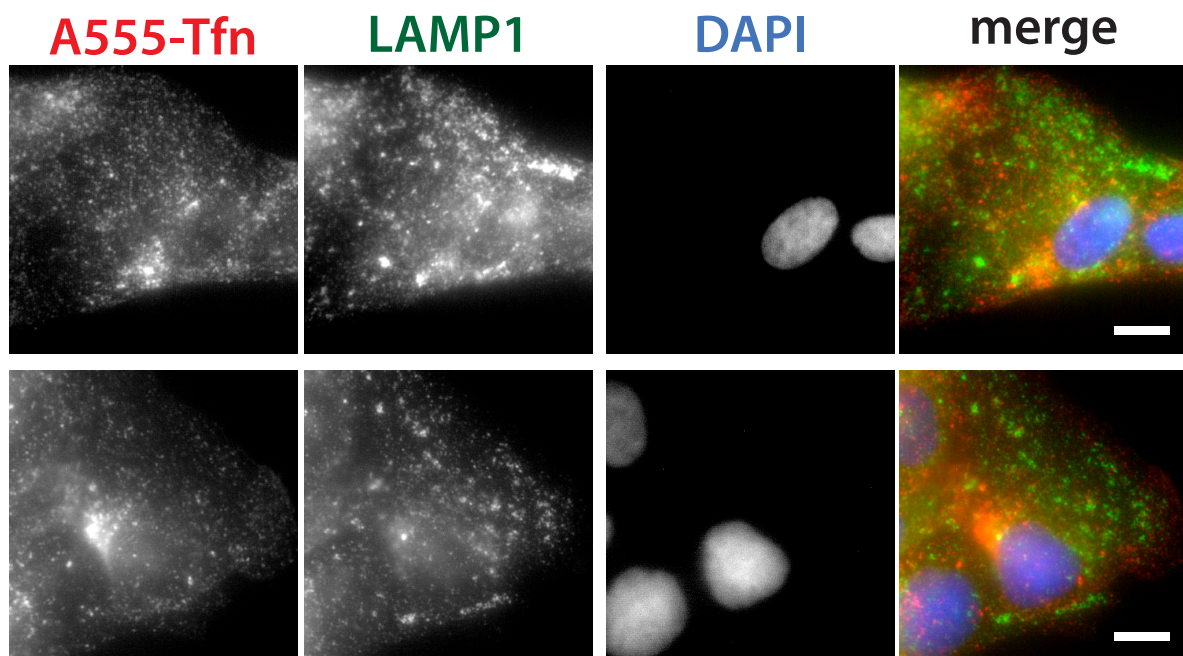

Supplement: Additional file 1: Figure S1. — Transferrin-loaded endosomes and LAMP-1 positive lysosomes are largely distinct compartments. RPE cells (not expressing any endogenous fluorescent proteins) were incubated with 10 μg/mL Alexa-555 conjugated transferrin (A555-Tfn) for 1 h at 37C. Subsequently, cells were rapidly washed, fixed, permeabilized, and subjected to immunofluorescence staining to detect endogenous LAMP-1. Cells were then further processed and subjected to widefield epifluorescence microscopy. Shown are two sets of representative micrographs of cells (under identical conditions) showing A555-Tfn (red), LAMP-1 (green), DAPI (blue) and merged images. These images showing little appreciable overlap of LAMP-1 and A555-Tfn signals. Scale = 10 μm. (PDF 4254 kb) [file 12896_2017_364_MOESM1_ESM.pdf]
